# Supplementary figures and images for: Antibody reactions of horses against various domains of the EHV-1 receptor-binding protein gD1
Source: PLoS One. 2024 Jul 12;19(7):e0301987. doi: 10.1371/journal.pone.0301987 (PMC11244823; doi:10.1371/journal.pone.0301987)

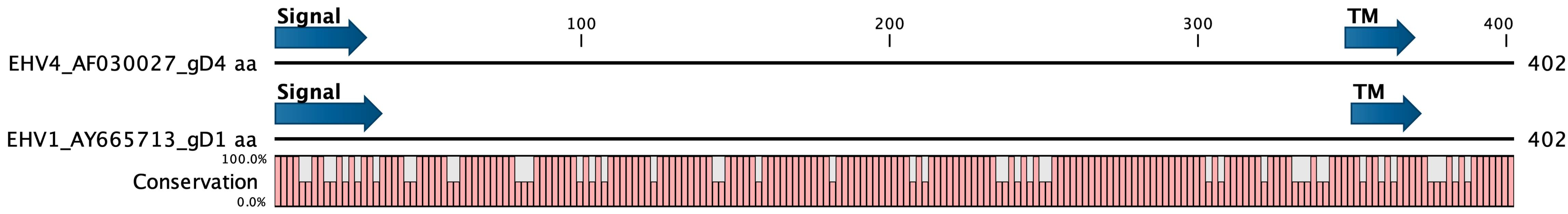

Supplement: S1 Fig — Signal peptide (Signal) and transmembrane regions (TM) are indicated. Amino acids are counted from left to right, starting with the amino terminus of the molecule. Full bars in the Conservation graph (red bars) indicate aa identity; half bars indicate differences. (TIFF) [file pone.0301987.s001.tiff]

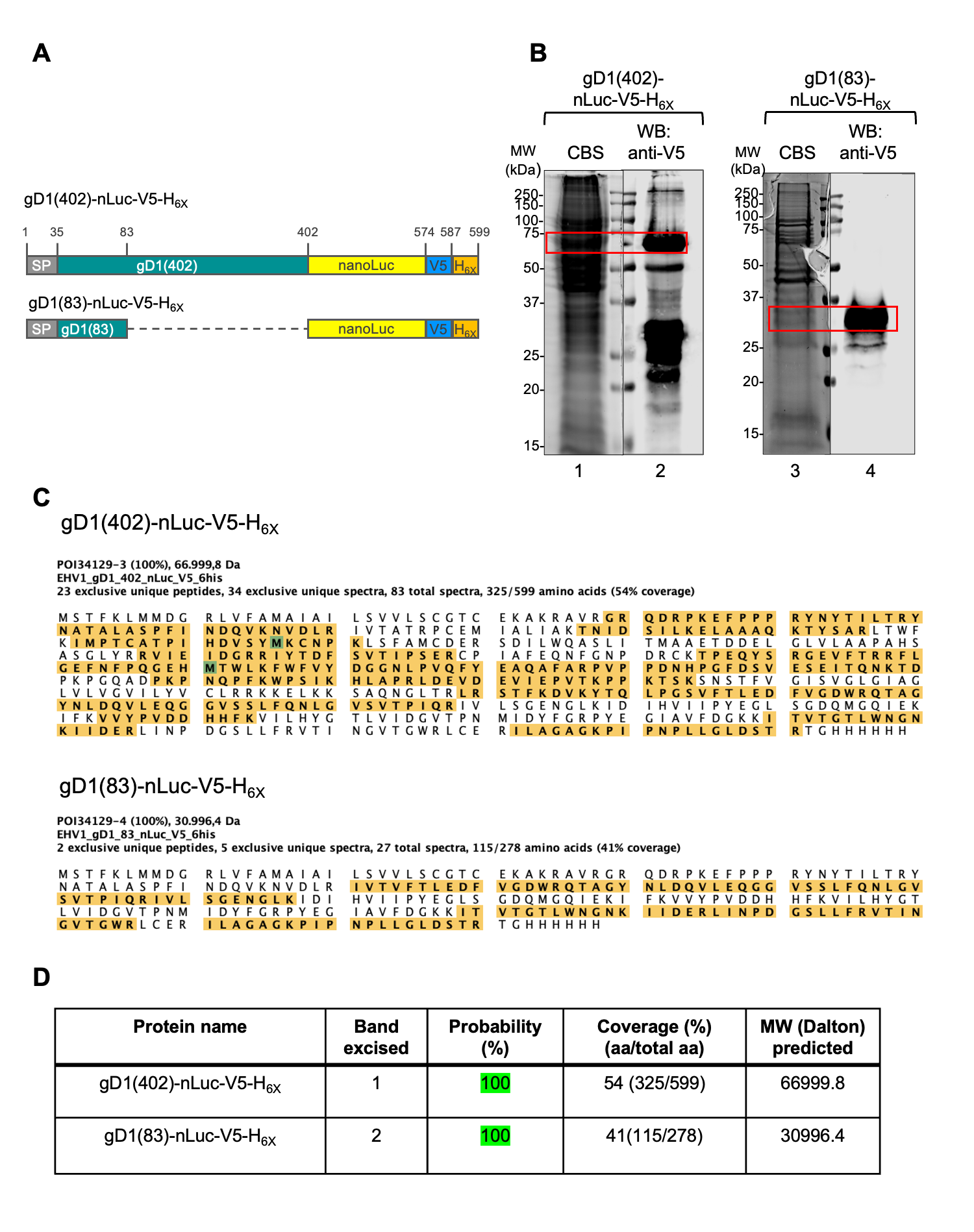

Supplement: S2 Fig — A) Schematic representation of gD1(402) and gD(83) fused to nanoLuc, V5 tag, and histidine tag (H6X), respectively. B) Coomassie blue staining (lanes 1 and 3) aligned with immunoblotting anti-V5 (lanes 2 and 4) of a cell extract of gD1(402)-nLuc-V5-H6X (left panel) and concentrated media supernatant of gD1(83)-nLuc-V5-H6X (right panel). C) Distribution of peptides coverage after MS analysis over the amino acid sequence of gD1(402)-nLuc-V5-H6X (upper panel) and gD1(83)-nLuc-V5-H6X (lower panel). The yellow highlighted amino acids correspond to the detected regions. D) Table indicating the probability (%), coverage peptide (%), and molecular weight for the indicated proteins. The green highlighted indicated 100% confidence in the result. (TIFF) [file pone.0301987.s002.tiff]

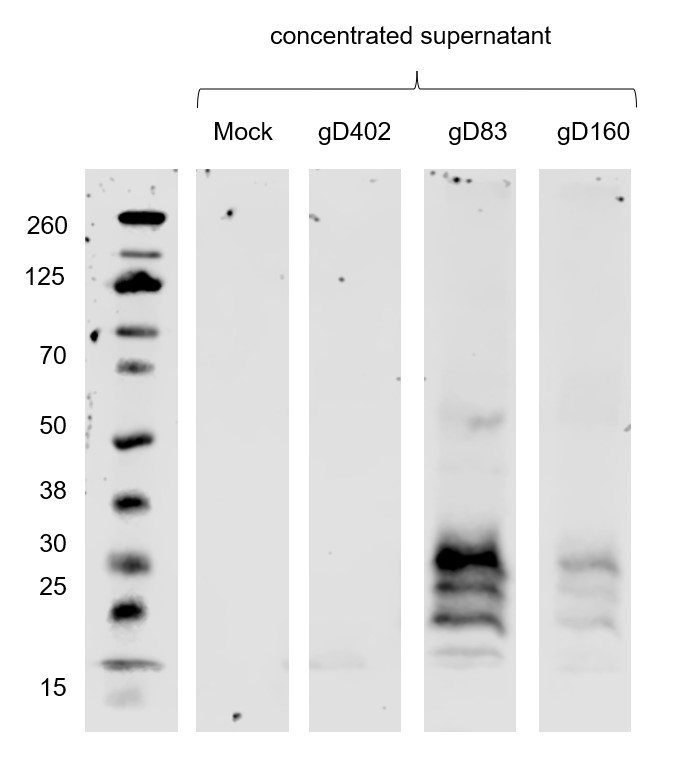

Supplement: S3 Fig — Apparent mobility: gD1_402 70 kDA, gD1_83 25kDa and gD1_160 38 kDA. (JPG) [file pone.0301987.s003.jpg]

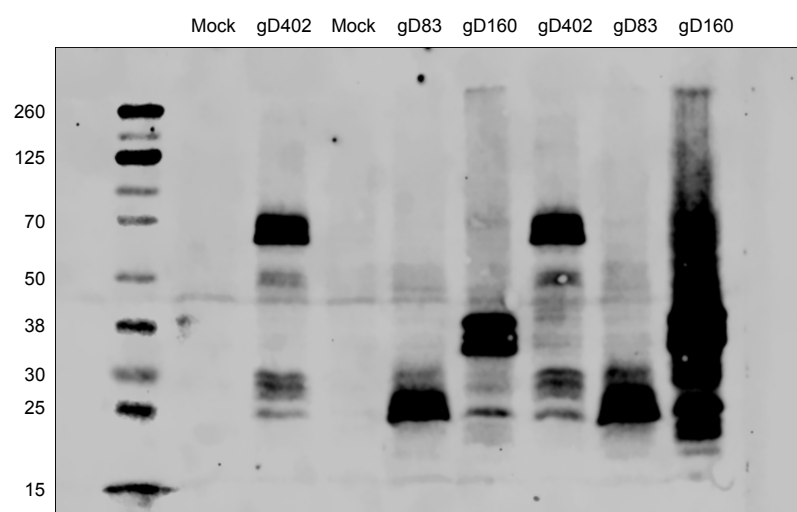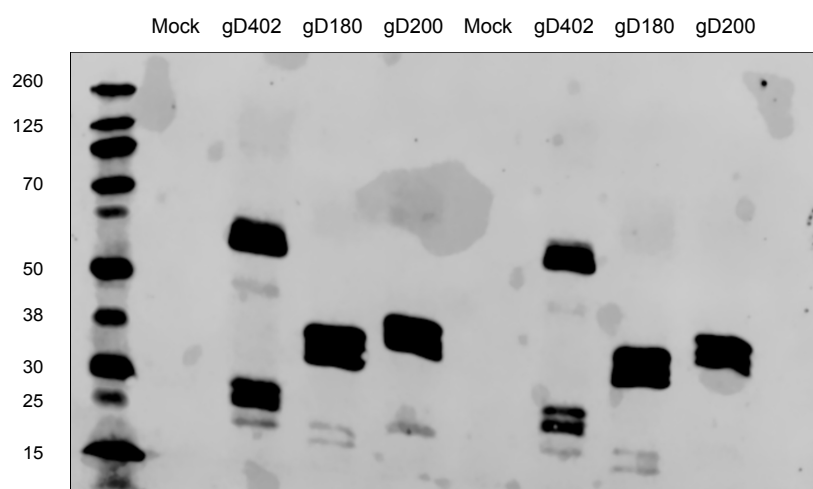

Supplement: S1 File — (PDF) [file pone.0301987.s004.pdf]
