# Supplementary material for: Antibody reactions of horses against various domains of the EHV-1 receptor-binding protein gD1
Source: PLoS One. 2024 Jul 12;19(7):e0301987. doi: 10.1371/journal.pone.0301987 (PMC11244823; doi:10.1371/journal.pone.0301987)
Supplement: Attachment — Submitted filename: Response to reviewers.docx [file pone.0301987.s006.docx]

**Response to Reviewers**

**Reviewers' comments:**

**Reviewer #1: Viviane Kremling** The work aims to identify regions of gD1 that give rise to type-specific antibodies after infection with EHV-1 and/or vaccination. Cross reactive antibodies against gD1 and gD4 are known but knowledge about type-specific gD1 antibodies is limited. For that purpose horse sera from Switzerland and from Iceland, which is considered EHV1-free, were used in a diagnostic ELISA and adapted Luciferase immunoprecipitation system (LIPS) assays. Three antigens consisting of N-terminal gD1 protein fragments plus the full-length protein were tested. Full length gD1 LIPS assay detected cross-reactive antibodies, however, gD1_83 fragment in LIPS assay seemed to identify gD1 specific antibodies in horse sera.

This work gives new insights into gD1 regions that can possibly give rise to type-specific antibodies which is important to understand the immune reaction after EHV-1 infection and vaccination. The results might help to improve vaccines which to date are not able to block an infection with either EHV-1 or EHV-4.
The data acquisition, assay validation, and statistical analysis seem sound. Weaknesses can be found in the interpretation of the data, placement into context of previous studies, and occasional spelling mistakes. I recommend the publication of this manuscript in PLOS ONE after revision.

| Reviewer’s comment | Authors’ comment |
| --- | --- |
| - line 57: “ EHV-1 in which the original gD (gD1) had been deleted and replaced by EHV-4 gD (gD4)” I am not sure that this statement of a deletion and replacement of gD is correct. Usually essential genes are not easily deleted in viruses. Please check and provide a reference. | *In REF 17, Azab et al. (2012) describe the procedure: Bac mutagenesis by deletion of gD in bacmid and insertion of heterologous gD amplified from plasmids 🡪 generation of recombinant virus after transfection of bac*  *In REF 18, Holz et al. (2017) describe the resulting phenotype in horses* |
| - line 60-61: On what data do you build your hypothesis that type-specific epitopes of gD1 could be important for protection against infection? | *We provided two references for our hypothesis:*  *REF 19, Edington & Bridges (1990): primary EHV-1 infection cross-protecting against EHV-4 challenge but primary EHV-4 infection not cross-protective against EHV-1 challenge and, interestingly, EHV-1 infection produced type-specific VN but type common CF antibody response whereas both VN and CF antibodies after primary EHV-4 infection were type specific.*  *REF 7, Patel & Heldens (2005): Equine herpesviruses 1 (EHV-1) and 4 (EHV-4) – epidemiology, disease and immunoprophylaxis: A brief review* |
| - line 70-71: Unfortunately the sentence is unclear and with that the aim. There is probably a typo in the beginning of line 71. I understand the sentence in a way that the aim is to identify gD1 fragments that bind type-specific antibodies that arise after infection. If that is so, I wonder why mostly sera from horses was used that were vaccinated as stated in line 464-465 even more so since it is also interesting to see if specific antibodies are formed after vaccination? | *The sentence was adapted.*  *For clarification: in the study, the majority of sera were collected from horses without vaccination history. In M&M lines 94 – 95 we state, that among the 60 Swiss horses, 40 did not and 20 did have a vaccination history.*  *In Iceland, vaccination against EHV-1 is prohibited, which is why all horses are classified as unvaccinated.*  *In Discussion lines 464 – 465, we do not state that the vaccinated horses were overrepresented, but that vaccinated horses were overrepresented in the group with positive reactions against gD83.*  *Line 105 – 106: it was added that vaccination is prohibited in Iceland* |
| - line 128, 131: You should choose one notation for the Cos cells. “Cos cells” or “COS-7 cells”. | *The spelling COS-7 was adopted throughout the manuscript.* |
| - line 151: You probably meant “comprising” instead of “compromising”. | *The typo has been corrected.* |
| - line 159: I am a bit confused about this sentence. Was the amino acid sequence of the proteins checked or the nucleic acid sequence of the transfected DNA and how? | *The sentence was adapted and moved to line 134.* |
| - line 254: There is an empty box next to the black filled square which probably should not be there. | *Thank you for the comment. This artifact emerged upon printing of our word file as pdf. Upon finalizing our manuscript for publication, we will keep this issue in mind.* |
| - line 280-283: Can you please comment on what influence glycosylation of the protein fragments could have on your assay. Would this influence the antibody binding? And if so, would this have any effect on your LIPS assay? | *Certainly glycosylation plays a role with regard to antibody-binding to a particular epitope. Under natural conditions in virus infections, both glycosylated and non-glycosylated forms of the same protein may be immunogenic, thus, potentially giving rise for antibodies against the glycosylated epitope as well as its non-glycosylated precursor. With a mixture of both antigenic forms in the detection system, these two forms cannot be discriminated. Indeed, this issue was not addressed in the present study but would be interesting to address in the future.* |
| - line 284-285: Since no gD1 specific antibodies were used in the western blot it might be helpful to verify the fragments by mass spectrometry. | *Thank you. We performed MS of gD402 cell lysate and gD83 supernatant in collaboration with FGCZ at the UZH. The identity of the antigens was confirmed in both cases with 100% probability.*  *We have added new sections in M&M (from line 176) and Results, including Supplementary Fig. 2 (from line 284)* |
| - line 291-293: The figure legend should include the notion of glycosylated gD. | *Glycosylated and non-glycosylated forms of antigen were marked in Figure 4.* |
| - line 411-412: You state that the gD1 fragments are thought to be secreted into the cell culture supernatant. Can you provide data for this claim? If I understood it right you used only cell lysate for western blotting where all protein fragments were apparently detected. It should be more clear if the presented data was generated with protein from cell lysate or supernatant. | *We added a supplementary figure (Supplementary Fig. 3) showing bands for gD1_83 and gD1_160 in the supernatant, whereas no bands are visible for gD1_402, which is supposed to remain cell associated due to its membrane anchor. gD1_180 supernatant was not tested.* |
| - line 414-417: It is not clear to me why it is a consequence from protein being secreted into cell culture supernatant or not in an artificial system that only full length gD1 will be recognized by cross-reactive antibodies. Can you please comment on that and provide references? | *The point is not whether it is secreted or not, but whether cross-reacting epitopes are present or not. In the case of complete molecules, such epitopes are certainly present; in the case of fragments, they may or may not be present.* |
| - line 417-420: Is the prediction stated here based on any other study or data? Why would the shortest fragment be the one which is most likely to carry the type-specific epitope? Is this based on structural data? | *See lines 65 – 69 including REFS 17, 19, 20* |
| - line 423-425: Do you have any idea why the gD1_160 did not work so well in your LIPS assay and why the positive sera values varied so much? | *A possible explanation was added.*  *See also new section “AlphaFold analysis” from line 376* |
| - line 442: How do you explain the one “true positive” result from an icelandic horse serum? | *The following sentence was added:*  *The true positive result in the Icelandic horse serum remains unexplained. The serum of this horse tested seronegative for gG1 and seropositive for gG4 by ELISA. Although Iceland is considered EHV-1 free and vaccination is prohibited, there were also EHV-1 positive sera in the ELISA, the origin of which is unknown.* |
| - line 458-462: For point 2 and 3 a references are needed. It would be helpful for the reader to have info about the kind of vaccines that are in use and the immune responses that are to be expected. | *The following references were included:*  *Point 2: REF 41, Allkofer et al., 2021; REF 40, Wagner et al., 2015*  *Point 3: REF 41, Allkofer et al., 2021; REF 2, Kydd et al., 2006* |
| - line 473: A reference is needed for the statement that type-common antibodies are not protective against EHM. | *We provided two references for the statement:*  *REF 7, Edington & Bridges, 1990: primary EHV-1 infection cross-protecting against EHV-4 challenge but primary EHV-4 infection not cross-protective against EHV-1 challenge and, interestingly, EHV-1 infection produced type-specific VN but type common CF antibody response whereas both VN and CF antibodies after primary EHV-4 infection were type specific.*  *REF 19, Patel & Heldens, 2005: Equine herpesviruses 1 (EHV-1) and 4 (EHV-4) – epidemiology, disease and immunoprophylaxis: A brief review* |
| - line 474: “It is uncertain that...” should be replaced by “It is uncertain if...”. | *The sentence was changed accordingly.* |
| - Figure 7 and Figure 9 are the same. | *Thank you for the remark. The correct figure 7 has been uploaded* |
| - It would be really interesting to try to link the data to the structure of gD1 and gD4 protein in the discussion. It should be taken into account that fragmenting of proteins can result in different molecular structures which can influence antibody binding. A quick modeling via AlphaFold might help to see, if the structure of the gD1 fragments can be expected to correspond to full gD1. | *Thank you for this input. AlphaFold analysis was performed, and new sections added in M&M (from line 176) and Results, including Fig. 9 (from line 376).* |

**Reviewer #2:** Overall:
In this paper the authors investigate antibody responses to the related equine herpesviruses (EHV1 & 4, which exhibit significant sequence similarity). Former can give rise to EHM. What disease/clinical signs does EHV4 elicit?
Serum antibodies were detected via immunoprecipitation assay, to the gD glycoprotein (GP) via a series of N terminal fragments of increasing length. Compared with ‘type-specific’ ELISA. Horse sera from two distant and distinct countries (Switzerland and Iceland) with different EHV incidence/vaccination.

Generally very well written, scientifically thorough and clear article.
Mostly very good, detailed description of M&M and Results.
Discussion raises some interesting points, some covered in a fulsome manner but other statements are not always followed up as fully as they could be – see recommendations in Minor Comments.
[Clarify if any EHV vaccination is carried out on Iceland – I presume not, as would impact study. I note brief mention in the M&M.]

| Reviewer’s comment | Authors’ comment |
| --- | --- |
| Abstract |  |
| Need to state Iceland was chosen as believed to be free of EHV1. | *Statement was added to line 22*. |
| Either Luciferase should be in lower case or immunoprecipitation and system in uppers case (for the LIPS abbreviation) and used throughout. | *“Luciferase” is spelled in lower case throughout the manuscript* |
| Introduction |  |
| Line 17 – insert ‘respectively’ after gG4 in (gG1 and gG4). | *The sentence was corrected accordingly.* |
| Line 50 – suggest noted that these alternative vaccines used outside Switzerland. | *The sentence was adapted accordingly.* |
| Line 68 – the region of non-identical amino acids should also be defined. | *The sentence was adapted and a supplementary figure (Suppl. Fig. 1) included for visualization.* |
| Line 75 – briefly explain A/G-coated bead system. | *The sentence was adapted accordingly.* |
| Line 85 – not that this would be due to cross-reactive antibodies. | *The sentence was adapted accordingly.* |
| Materials and Methods |  |
| Line 128 – give source and variant # of COS cells and how these were codon optimized (as they are not human cells). | *Source of COS cells was added to line 136.*  *For clarification: not COS cells but desired aa sequences were reverse-translated to nucleic acid sequence, which were then codon optimized. This was done by GenScript (see lines 127 – 129)* |
| Line 129 – define cmv (also should be upper case - CMV) | *Sentence was adapted accordingly.* |
| Line 148 – ‘scrapped’ should read ‘scraped’. | *Typo was corrected.* |
| Line 158/159 – sequencing line should be clarified – maybe ‘coding region’ rather than protein. | *Sentence was adapted and moved to line 134.* |
| Line 169 – ‘skimmed milk’. | *Typo was corrected*. |
| Line 217 – mention Graphpad software company. | *The sentence was adapted accordingly.* |
| Results |  |
| Line 245 – It is stated that 21 Swiss and 7 Icelandic sera were gG1 positive (a type-specific ELISA test), however it is then stated that ‘a history of EHV1 was only rarely confirmed’ – this is hardly ‘rare’. Rephrase – lower levels than EHV4 detection? | *Rephrased to “less frequently”* |
| Line 263-267 are methods rather than results, so should be moved to M&M section. | *Since all the information is already provided in M&M, lines 263 – 267 were deleted.* |
| Line 268 – should read 1-6x. | *Adapted accordingly* |
| Line 270 – the Fig 3 data for gD1_180 and 402 should be mentioned (for discussion later) as they are interestingly higher than for 160 (noting 160 also gave lower sensitivity result in Fig 5, as mentioned in later Results). | *The sentence was adapted accordingly.* |
| Line 344 – clarify ‘with one exception’. | *“negative control serum No. 62” was added in brackets.* |
| Line 345 – rephrase ‘clouded’. | *Changed to “clustered”.* |
| Discussion |  |
| Line 403 – whole sentence needs clarifying – from production to transport to secretion (during infection or plasmid transfection or both?). What is cleaved off (and by which protease?). Is the 48aa truly a fragment or a section? | *The sentence was adapted, and a reference added (REF 39, Wellington et al., 1996)* |
| Line 423 – authors should discuss why the gD_160 should give such poor responses and yet even 180 gives a reasonable response. | *See new section “AlphaFold analysis” from line 376* |
| Line 435 – Authors should discuss this discrepancy – were different test assays used? Is monitoring often conducted. Do horses travel internationally from Iceland to events/breeding? | *The sentence was adapted.* |
| Line 462 – explain why you might see a preference in antibody response against the different antigens in different animals – is this referring to differences between vaccination and natural infection? | *See comment to reviewer 1:*  *The following references were included:*  *REF 41, Allkofer et al., 2021; REF 40, Wagner et al., 2015, REF2, Kydd et al., 2006* |
| Line 472 – are the authors referring to ‘antigenic sin’? If so, how might this impact type-specific vaccination as suggested at the end of the Discussion? | *Yes, a reference (REF 42, Vatti et al., 2017) referring to this issue was added.* |
